# Associations of protein source, distribution and healthy dietary pattern with appendicular lean mass in oldest-old men: the Helsinki Businessmen Study (HBS)

**DOI:** 10.1007/s41999-020-00330-1

**Published:** 2020-05-22

**Authors:** S. K. Jyväkorpi, A. Urtamo, M. Kivimäki, T. E. Strandberg

**Affiliations:** 1 University of Helsinki, Clinicum and Helsinki University Central Hospital, Unit of Primary Health Care, Tukholmankatu 8 B, 00014 Helsinki, Finland; 2 University of Helsinki, Clinicum, and Helsinki University Hospital, Helsinki, Finland; 3 University of Oulu, Center for Life Course Health Research, Oulu, Finland

**Keywords:** Appendicular lean mass, Protein intake, Protein distribution, Protein source, Fruits and vegetables, Animal protein, Meat intake

## Abstract

**Purpose:**

We explored how food and dietary intakes, protein daily distribution and source are associated with appendicular lean mass (ALM)/m^2^ of the oldest-old community-dwelling men.

**Methods:**

Cross-sectional analyses of Helsinki Businessmen Study (HBS, mean age 87 years) participants who came to clinic visit in 2017/2018. Nutritional status, physical performance and fasting blood samples were measured. Food and dietary intakes were retrieved from 3-day food diaries. Body composition was measured and appendicular lean mass (ALM) per m^2^ was dichotomized as ALM/m^2^ < 7 kg/m^2^ and ≥ 7 kg/m^2^. Differences between lower and higher ALM were analyzed using *t* test or Mann–Whitney *U* test. Analysis of covariance was used to investigate independent associations with ALM/m^2^.

**Results:**

Random sample of 130 participants took part in the medical examinations, 126 returned food diaries, and 102 underwent DXA-scan. ALM/m^2^ was associated with total protein (*p* = 0.033), animal protein (*p* = 0.043) and meat protein (*p* = 0.033) intakes. Protein distribution between daily meals differed at lunch; those with higher ALM/m^2^ ate more protein (p = .047) at lunch. Consumption of fruits, vegetables (*p* = 0.022) and meat (*p* = 0.006) was associated with ALM/m^2^.

**Conclusion:**

Protein intake, source and distribution as well fruit and vegetable intakes were associated with higher ALM in oldest-old men.

**Study registration:**

The study is registered with ClinicalTrials.gov identifier: NCT02526082.

## Key summary points

**Aim:**

To investigate how food and dietary intakes, protein daily distribution and source were associated with appendicular lean mass (ALM)/m^2^ in the oldest-old community-dwelling men.

**Findings:**

ALM/m^2^ was associated with total protein intake, source and distribution as well as fruit and vegetable intakes.

**Message:**

Not only protein intake, but also source and distribution as well as healthy overall diet characterized by abundant amounts of fruits and vegetables were important in maintaining muscle mass in the oldest-old men in our study.

## Introduction

Skeletal muscle is highly important for metabolic health and maintenance of physical function in older age [1]. Skeletal muscle mass and strength decline steadily after the fourth decade of life and the rate of decline are accelerated with aging [2]. Loss of skeletal muscle mass is an independent risk factor for osteoporosis, falls and fractures, impaired functioning and mortality [3].

Skeletal muscle is also a major organ of glucose metabolism and thus low skeletal muscle may impair glucose tolerance and insulin resistance [4]. For these reasons, there has been a great interest to define lifestyle-related risk factors of skeletal muscle loss. Of nutritional factors, especially inadequate protein intake has been associated with accelerated loss of lean mass, and an increased risk of functional impairments, whereas adequate protein intake has been linked to muscle protein balance and slower rate of muscle mass decline [5]. Higher protein intake has also been associated with increase in muscle mass especially in relation to exercise [6]. Of dietary patterns, Mediterranean diet has been positively associated with muscle mass in older adults [7, 8]. However, data on the fastest growing age group of the oldest old (> 85 years) are very limited with respect to nutrition and muscle mass.

To address this limitation, we explored detailed food and nutrition intakes, distribution of daily protein intake and source between lower and higher appendicular lean mass (ALM) groups in oldest old, community-dwelling men.

## Methods

In the Helsinki Businessmen Study (HBS) socioeconomically homogenous cohort of men, born between 1919 and 1934, have been followed-up since the 1960s [9]. In the present cross-sectional analysis, we report findings from the most recent clinic visit including a random sub-cohort of home-living survivors of HBS in 2017–2018 (mean age 87 years of age). At the clinic visit, body mass index (BMI) was calculated as weight (kg/height (m) squared), Mini Nutritional Assessment (MNA) [10] and Short Physical Performance Battery (SPPB) [11] were performed as instructed, body composition measured with DXA-scans, and appendicular lean mass (ALM) per m^2^ was calculated. ALM/m^2^ was dichotomized as < 7 kg/m^2^ and ≥ 7 kg/m^2^ according to the classification of Gould et al. [12]. Blood insulin and glucose levels were analyzed from blood samples after the 12 h fast. Food, energy and nutrient intakes, daily protein distribution and protein source (amounts of vegetable, animal; milk, meat, fish, and egg proteins) were calculated from the 3-day food diaries.

Statistical significance for group differences was evaluated using independent t test for evenly distributed continuous variables and Mann–Whitney U test for unevenly distributed variables. In addition, we used analysis of covariance (ANCOVA) to investigate independent associations with ALM/m^2^. Adjustments were made for age, BMI, protein intake, g, insulin levels and tea drinking. Analyses were performed using the SPSS statistical program, version 24 (SPSS IBM, Armonk, NY, USA).

### Selection of covariates

Covariates were selected based on the results of our analysis and prior research. Insulin was selected as a covariate because muscle is a major organ for glucose metabolism [4], tea because it was inversely associated with higher ALM in t test, and protein intake for its importance to muscle. Age is associated with loss of skeletal muscle mass and BMI with higher muscle mass.

## Results

130 men participated in the clinic visit, 126 returned food diaries and 102 underwent the DXA scan. Age, MNA, SPPB or insulin and glucose levels did not differ significantly between the two ALM/m^2^ groups. Higher ALM/m^2^ was associated with higher BMI (*p* < 0.001), total protein intake (*p* = 0.033), consumption of protein of animal origin (*p* = 0.043) and meat protein (*p* = 0.033). Protein distribution between daily meals differed at lunch only; those in the higher ALM/m^2^ group ate 26 g protein at lunch, compared to 20 g in those with low ALM/m^2^ (*p* = 0.047, Table 1). Of foods consumed, total fruits and vegetables intakes differed significantly between the ALM/m^2^ groups; those with higher ALM/m^2^ consumed more fruits and vegetables: 341 g/d compared to 243 g/d (*p* = 0.022, Table 2). Meat intake was higher in those who had higher ALM/m^2^ (*p* = 0.006), whereas tea intake was negatively associated with ALM/m^2^ (*p* = 0.027).

**Table 1:** Baseline characteristics, protein distribution between daily meals and protein source by level of appendicular lean mass (ALM)/m^2^

Baseline characteristics	ALM groups		
	ALM < 7 kg/m^2^ *n* = 45	ALM ≥ 7 kg/m^2^ *n* = 57	*p* value^a^
Age, years	87 (3)	87 (3)	0.670
BMI, kg/m^2^	24.7 (2)	26.6 (3)	< 0 .001
MNA points (range 0–14) (SD)	13 (1)	13 (1)	0.249
SPPB points (range 1–12) (SD)	9 (3)	10 (2)	0.215
Insulin, mmol/L	8.8 (5.1)	7.3 (3.3)	0.112
Glucose, mmol/L	6.2 (1.0)	6.2 (0.8)	0.905
Protein distribution between daily meals, (g)			
Breakfast, g (SD)	16 (8)	16 (7)	0.927
Morning snack, g (SD)	2 (3)	4 (8)	0.071
Lunch, g (SD)	20 (12)	26 (15)	0.047
Dinner, g (SD)	20 (20)	19 (16)	0.692
Afternoon snack, g (SD)	5 (8)	5 (7)	0.615
Evening snack, g (SD)	6 (8)	8 (7)	0.141
Total Protein, g (SD)	69 (24)	79 (21)	0.033
Protein % of total energy	19.4% (3.3)	17.8% (3.3)	0.016
Protein source, g			
Animal protein total, g (SD)	48 (21)	56 (18)	0.043
Meat protein, g (SD)	19 (12)	24 (13)	0.033
Milk protein, g (SD)	16 (10)	18 (10)	0.374
Egg protein, g (SD)	2 (3)	2 (3)	0.943
Fish protein, g (SD)	11 (12)	12 (11)	0.717
Plant protein, g (SD)	21 (7)	22 (7)	0.373
Total protein, g (SD)	69 (24)	79 (21)	0.033

*BMI* body mass index, *MNA* Mini Nutritional Assessment, *SPPB* short physical performance battery, *SD* standard deviation, *ALM* appendicular lean mass

^a^Difference between higher and lower ALM was tested with independent *t* test in even distributed variables and Mann–Whitney *U* test for unevenly distributed variables

**Table 2:** Food, energy and nutrient intake by level of appendicular lean mass (ALM)/m^2^

Food intake/day	ALM groups		
	ALM/m^2^ < 7 kg *n* = 45	ALM/m^2^ ≥ 7 kg *n* = 57	*p* values^a^
Fruits and berries, g (SD)	112 (119)	170 (183)	0.056
Vegetables, g (SD)	138 (104)	176 (153)	0.163
Total fruits and vegetables, g (SD)	243 (163)	341 (265)	0.022
Whole grain products, g (SD)	98 (60)	102 (65)	0.738
Other grain products, g (SD)	233 (130)	242 (148)	0.749
Legumes, g (SD)	9 (24)	6 (18)	0.448
Nuts, g (SD)	1 (3)	8 (20)	0.060
Fish, g (SD)	56 (56)	66 (61)	0.364
Milk products, g (SD)	280 (222)	347 (220)	0.138
Meat, g (SD)	89 (48)	120 (60)	0.006
Egg, g (SD)	17 (30)	15 (26)	0.761
Alcohol, g (SD)	7 (10)	4 (7)	0.136
Tea, g (SD)	87 (141)	160 (177)	0.027
Coffee, g (SD)	218 (159)	294 (216)	0.051
Energy and nutrient intakes			
Energy, kcal (SD)	1550 (391)	1634 (358)	0.262
Protein, g (SD) g/kg BW/d	69 (24) 0.95 (0.3)	80 (21) 0.99 (0.24)	0.033 0.584
Carbohydrates, g (SD)	164 (44)	170 (43)	0.505
Fat, g (SD)	63 (21)	67 (22)	0.314
Vitamin D, µg (SD)	9 (8)	10 (7)	0.310
Vitamin E, mg (SD)	10 (4)	11 (6)	0.181
Iron, mg, (SD)	10 (3)	11 (3)	0.174

*BMI* body mass index, *SD* standard deviation, *ALM* appendicular lean mass

^a^Difference was between higher and lower ALM was tested with independent t test

General linear model confirmed the bivariate findings with respect to protein intake and BMI to ALM/m^2^. Protein intake remained significant to ALM/m^2^ after adjusting for age, and additionally with insulin levels, BMI, and tea intake (Table 3).

**Table 3:** ANCOVA models of factors associated with appendicular lean mass (ALM)/m^2^

		95% confidence interval		
	*B*	Lower bound	Upper bound	*p* value
Model 1				
Intercept	9.724	5.641	13.807	< 0.001
Age	− 0.038	− 0.085	0.009	0.111
Protein, g	0.009	0.003	0.015	0.003
Adjusted *R*^2^	**0.087**			
Model 2				
Intercept	5.205	1.161	9.249	0.012
Age	− 0.018	− 0.060	0.025	0.406
Protein, g	0.005	− 0.026	0.825	0.067
BMI	0.119	0.073	0.165	< 0.001
Adjusted *R*^2^	**0.273**			
Model 3				
Intercept	5.368	1.379	9.357	0.009
Age	− 0.018	− 0.060	0.024	0.392
Protein g	0.005	0.000	0.011	0.063
BMI	0.124	0.078	0.169	< 0.001
Insulin	− 0.033	− 0.061	− 0.005	0.020
Adjusted *R*^2^	**0.300**			
Model 4				
Intercept	5.368	1.437	9.299	0.008
Age	− 0.017	− 0.058	0.024	0.420
BMI	0.120	0.099	0.185	< 0.001
Protein, g	0.418	0.076	0.165	0.048
Insulin	− 0.030	− 0.056	0.003	0.032
Tea, g/d	− 0.001	− 0.001	< 0.000	0.052
Adjusted *R*^2^	**0.320**			

Bold values indicate the amount of observed variation that can be explained by the model’s inputs

*B* unstandardized beta, *BMI* body mass index, *BW* body weight, *kg* kilograms, *g* grams, *d* day

## Discussion

In this study, total protein intake, protein source and distribution as well as meat, fruit and vegetable intakes were associated with higher ALM/m^2^ in oldest old community-dwelling men, whereas tea drinking was inversely associated with ALM/m^2^. Only participants in the higher ALM/m^2^ group reached the amount of protein in a single meal considered to be sufficient for effective protein synthesis.

Proteins of animal origin are high in essential amino acids important for the muscle. Especially the amino acid leucine that is abundant in foods of animal origin has been shown to be important for muscle development and strength [13]. Therefore, it was not surprising that meat protein and meat consumption were associated with higher ALM/m^2^ in our study. Earlier studies have suggested that even protein distribution in daily meals would be most beneficial for older people in relation to muscle health [14]. However, meals with high protein bolus have also been found to be beneficial [15]. In our study protein distribution was relatively even in both ALM groups, as breakfast, lunch, and dinner were the meals with the highest protein intake, whereas the daily snacks contained relatively low amounts of protein. It has been suggested that ingestion of approximately 25–30 g of protein per meal maximally stimulates muscle protein synthesis in older people [13]. Only the participants classified under the higher ALM group reached this amount in a single meal at lunch, which was the only meal that differed significantly between the ALM groups in our study. The lower ALM group did not reach this threshold in any of their daily meals. These findings underline the importance of educating older people about timing and distribution of their protein intake.

Those with higher ALM ate more fruits and vegetables, which are an essential part of several healthy dietary patterns, including Mediterranean, Nordic, and DASH diets. Fruits and vegetables contain high amounts of vitamins, minerals, antioxidants, and other bioactive compounds and in addition, alkaline salts may also be important in preserving muscle [7, 8, 15]. Tea drinking was associated with lower ALM/m^2^, but as we did further testing, we found that tea drinking was associated with lower BMI which would explain the result.

The strengths of this study include its robust findings—despite the relatively small study sample—and the fact that there are few other studies on oldest-old (> 85 years of age) people. Limitations involve generalization: the survivors of HBS differ in many ways from the general population by being men from upper socioeconomic class; and the cross-sectional design of the study, which prevents drawing conclusions about causal relationships.

## Conclusions and implications

Our study extends previous findings on the importance of protein intake and the threshold in a single meal in the maintenance of muscle mass of the oldest-old. Moreover, fruit and vegetable intakes, emphasized in healthy dietary patterns, were also important for muscle mass. Therefore, the importance of protein intake and distribution, as well as healthy dietary patterns should be highlighted also for the oldest-old.

## Data Availability

The data can be enquired from Professor Timo Strandberg, timo.strandberg@oulu.fi.

## References

[1] Tieland M, Trouwborst I, Clark BC (2018). Skeletal muscle performance and ageing. J Cachexia Sarcopenia Muscle.

[2] Roshanravan B, Patel KV, Fried LF, Robinson-Cohen C, de Boer IH, Harris T (2017). Association of muscle endurance, fatigability, and strength with functional limitation and mortality in the health aging and body composition study. J Gerontol A Biol Sci Med Sci.

[3] Johnson ML, Robinson MM, Nair KS (2013). Skeletal muscle aging and the mitochondrion. Trends Endocrinol Metab.

[4] Scott D, de Courten B, Ebeling PR (2016). Sarcopenia: a potential cause and consequence of type 2 diabetes in Australia's ageing population?. Med J Aust.

[5] Houston DK, Nicklas BJ, Ding J, Harris TB, Tylavsky FA, Newman AB (2008). Dietary protein intake is associated with lean mass change in older, community-dwelling adults: the Health, Aging, and Body Composition (Health ABC) Study. Am J Clin Nutr.

[6] Tieland M, Dirks ML, van der Zwaluw N (2012). Protein supplementation increases muscle mass gain during prolonged resistance-type exercise training in frail elderly people: a randomized, double-blind, placebo-controlled trial. J Am Med Dir Assoc.

[7] Tyrovolas S, Haro J-M, Mariolis A, Piscopo S, Valacchi G, Bountziouka V, Anastasiou F, Zeimbekis A, Tyrovola D, Foscolou A (2016). Skeletal muscle mass and body fat in relation to successful ageing of older adults: the multinational MEDIS study. Arch Gerontol Geriat.

[8] Kelaiditi E, Jennings A, Steves C, Skinner J, Cassidy A, MacGregor A, Welch A (2016). Measurements of skeletal muscle mass and power are positively related to a Mediterranean dietary pattern in women. Osteoporos Int.

[9] Strandberg TE, Salomaa V, Strandberg AY (2016). Cohort profile: the Helsinki Businessmen Study (HBS). Int J Epidemiol.

[10] Vellas B, Guigoz Y, Garry PJ (1999). The Mini Nutritional Assessment (MNA) and its use in grading the nutritional state of elderly patients. Nutrition.

[11] Guralnik JM, Simonsick EM, Ferrucci L, Glynn RJ, Berkman LF, Blazer DG (1994). A short physical performance battery assessing lower extremity function: association with self-reported disability and prediction of mortality and nursing home admission. J Gerontol.

[12] Gould H, Brennan SL, Kotowicz MA (2014). Total and appendicular lean mass reference ranges for Australian men and women: the Geelong osteoporosis study. Calcif Tissue Int.

[13] Nowson C, O'Connell S (2015). Protein requirements and recommendations for older people: a review. Nutrients.

[14] Bouillande CE, Hamon-Vilcot B (2013). Impact of protein pulse feeding on lean mass in malnourished and at-risk hospitalized elderly patients: a randomized controlled trial. Clin Nutr.

[15] Dawson-Hughes B, Harris SS, Ceglia L (2008). Alkaline diets favour lean tissue mass in older adults. Am J Clin Nutr.

